# Systems-Level Analysis of HPAI H5N1 Infection in Ducks: Integrating Transcriptomic, Proteomic, and Phosphoproteomic Data

**DOI:** 10.3390/ijms27062884

**Published:** 2026-03-23

**Authors:** Periyasamy Vijayakumar, Anamika Mishra, Kandasamy Rajamanickam, Ashwin Ashok Raut

**Affiliations:** 1 Pathogenomics Laboratory, WOAH Reference Lab for Avian Influenza, ICAR-National Institute of High Security Animal Diseases, Bhopal 462022, Madhya Pradesh, India; nayaganviji@gmail.com (P.V.); reach2anamika@yahoo.com (A.M.); 2 Veterinary College and Research Institute, Tamil Nadu Veterinary and Animal Sciences University, Salem 636112, Tamil Nadu, India; rajamanickam.k@tanuvas.ac.in

**Keywords:** transcriptomics, proteomics, phosphoproteomics, avian influenza, ducks, pathways, hub genes

## Abstract

Ducks, once considered mere reservoirs, now serve as both victims and amplifiers of persistent highly pathogenic avian influenza (HPAI) virus cycles in wild populations. The molecular pathogenesis of HPAI is shaped by complex, dysregulated molecular networks, necessitating a systems biology approach that integrates computational modeling of host–pathogen interactions. Despite recent advances, a comprehensive understanding of the signaling pathways, molecular mechanisms, and hub genes driving HPAI H5N1 pathogenesis in avian hosts remains incomplete. This study addresses this gap by employing an integrated multi-omics strategy—combining transcriptomic, proteomic, and phosphoproteomic analyses—to map the signaling networks and key host factors involved in HPAI H5N1 infection in duck lung tissue. Our network analysis revealed activation of RIG-I-like receptor, toll-like receptor, NOD-like receptor, NF-κB, and JAK/STAT signaling pathways. Phosphoproteomic profiling independently confirmed the activation of these pathways, supporting the integrated network findings. Key regulatory hub genes identified include *STAT1*, *DDX58 (RIG-I)*, *MYD88*, *NFKBIA*, *NFKB1*, *IRF7*, *SOCS3*, *ACTB*, *TLR4*, *TLR7*, *IL-6*, *CASP1*, *and CASP8*, which form a central hub in duck antiviral immunity. Some of these genes may represent promising targets for therapeutic or vaccine development against avian influenza. Collectively, this work delineates the critical signaling pathways and hub genes underlying HPAI H5N1 pathogenesis in ducks through comprehensive multi-omics integration.

## 1. Introduction

Wild aquatic birds—including species such as ducks, geese, and swans from the order Anseriformes—are recognized as the primary natural reservoirs for avian influenza viruses (AIVs) [1,2]. Typically, these wild birds demonstrate innate resistance to highly pathogenic avian influenza viruses (HPAIVs), exhibiting either no symptoms or only mild clinical signs upon infection [3]. Nonetheless, certain Eurasian–African H5N1 HPAIV lineages, specifically clade 2.3.4.4, have been shown to induce systemic illness and high mortality rates in ducks under both natural and laboratory conditions [4,5,6,7]. The recent emergence and widespread dissemination of the H5N1 clade 2.3.4.4b virus have shifted this paradigm substantially. Ducks now function not solely as asymptomatic reservoirs but also as susceptible hosts and amplifiers, fueling ongoing cycles of HPAI virus transmission in natural environments [2]. Consequently, ducks occupy an indispensable and dynamic role in the ecology and evolutionary trajectory of AIVs.

The pathogenesis of infectious disease is a non-linear process, arising from dysregulated molecular networks characterized by dynamic feedback loops, post-translational modifications, and host–environment interactions. To decipher this complexity, systems biology offers a paradigm shift from reductionist studies of individual components to the computational modeling of the entire host–pathogen system as an integrated network. This holistic framework is crucial for achieving a predictive, mechanistic understanding of the emergent properties that govern infection outcomes. A cornerstone of this approach is the multi-omics integration of transcriptomic, proteomic, and phosphoproteomic data, which provides a multi-scale view of the disease process. Ultimately, the insights derived from systems-level models are instrumental for translating basic research into actionable interventions—such as the identification of novel therapeutic targets and the rational design of next-generation vaccines—thereby enhancing animal health, ensuring food security, and mitigating global pandemic threats [8,9,10].

Transcriptome analysis represents a widely accessible, high-throughput omics technology that frequently serves as a proxy for estimating protein abundance. In ducks, transcriptomic profiling using microarray and RNA sequencing methodologies has been employed to characterize innate immune responses elicited by various avian influenza virus infections [11,12,13,14,15,16,17]. Nevertheless, virus–host interactions are inherently multi-layered, and transcriptomic data captures only a subset of the host cellular responses. Thus, complementary high-throughput omics approaches, such as proteomics and phosphoproteomics, offer added dimensions of understanding disease pathogenesis by detailing post-translational modifications and signaling events [8,9,10]. Despite emerging efforts, avian proteomic studies related to influenza remain limited [18,19,20], and comprehensive elucidation of the key signaling pathways, molecular mechanisms and key host determinants underpinning highly pathogenic avian influenza (HPAI) H5N1 pathogenesis in avian hosts is still lacking. This study aims to address this knowledge gap by employing an integrated multi-omics approach, combining transcriptomic, proteomic, and phosphoproteomic data to characterize the signaling networks and critical host factors underlying HPAI H5N1 pathogenesis in duck lung tissue.

## 2. Results

**Figure 1:**
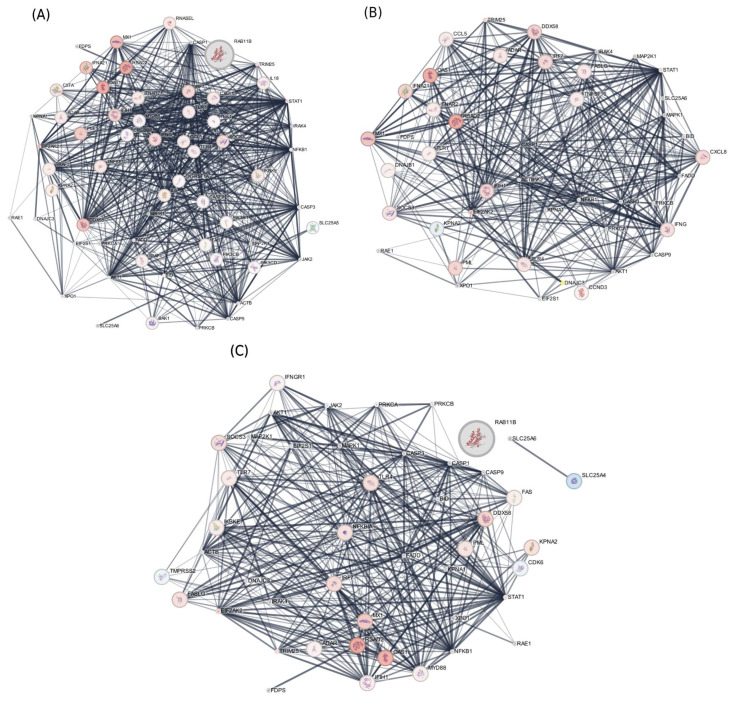
A protein–protein interaction (PPI) network generated using integrated transcriptomic and proteomic data from duck lung tissue at 12 h (**A**), 48 h (**B**), and 5 days (**C**) after HPAI H5N1 infection. Node color indicates mRNA expression changes (red for upregulation, blue for downregulation), while node size represents the magnitude of protein abundance changes (log2 fold change).

**Figure 2:**
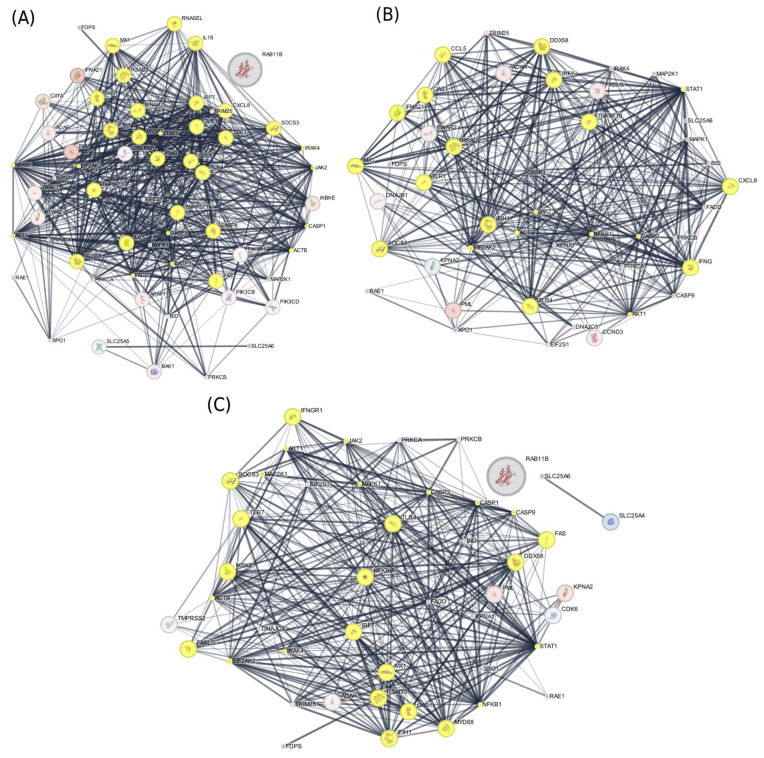
Clusters in the integrated transcriptomic and proteomic protein–protein interaction (PPI) network detected at 12 h (**A**), 48 h (**B**), and 5 days (**C**) post-infection using MOCDE software v1.0, with cluster nodes visually highlighted in yellow. Node color indicates mRNA expression changes (red for upregulation, blue for downregulation), while node size represents the magnitude of protein abundance changes (log2 fold change).

**Figure 3:**
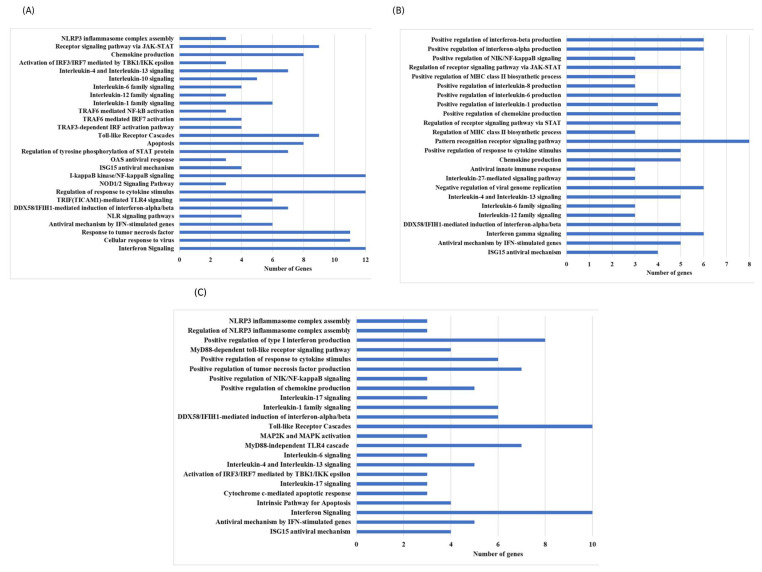
Analysis of clustered nodes from combined transcriptomic and proteomic PPI networks in duck lungs at 12 h (**A**), 48 h (**B**), and 5 days (**C**) after HPAI H5N1 infection identified enriched biological pathways relevant to disease immune response.

In this research, transcriptomic, proteomic, and phosphoproteomic data were integrated to investigate the innate immune responses of ducks infected with HPAIV H5N1, identifying key pathways involved in their antiviral defense. At 12 h post-infection, cluster and pathway enrichment analyses of the protein–protein interaction network (Figure 1 and Figure 2) revealed activation of toll-like receptor cascades, NOD-like receptor (NLR) signaling, DDX58/IFIH1-driven interferon-alpha/beta production, TRAF6-mediated IRF7 and NF-κB activation, I-kappaB kinase/NF-κB signaling, JAK-STAT receptor signaling, and interferon pathways (Figure 3). Similar analyses at 48 h and 5 days post-infection showed enrichment of chemokine production, cytokine response regulation, interleukin-12, -6, -4, and -13 signaling, and antiviral mechanisms mediated by interferon-stimulated genes (ISGs) (Figure 3). By 5 days post-infection, there was further enrichment of chemokine and cytokine responses, type I interferon production, apoptosis pathways, tumor necrosis factor (TNF) production, and ISG15-mediated antiviral mechanisms. These findings indicate that ducks activate pattern recognition receptor pathways—including TLRs, RLRs, and NLRs—which, in turn, stimulate transcription factors such as IRF7/IRF3 and NF-κB, leading to the production of type I interferons and pro-inflammatory cytokines and establishing a strong antiviral state (Figure 3).

**Figure 4:**
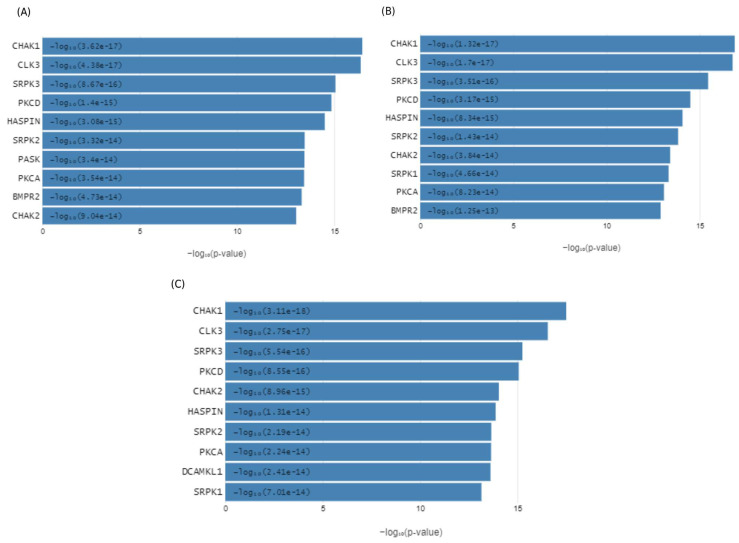
The ten most prominent upstream kinases, whose likely substrates are enriched among differentially phosphorylated proteins (*p* < 0.05), identified at 12 h (**A**), 48 h (**B**), and 5 days (**C**) following HPAI H5N1 infection in ducks. These kinases, mainly from CHAK1, CLK3, SRPK3, and PKCD kinases, are predicted to be activated during infection, reflecting dynamic changes in host phosphorylation signaling over time.

**Figure 5:**
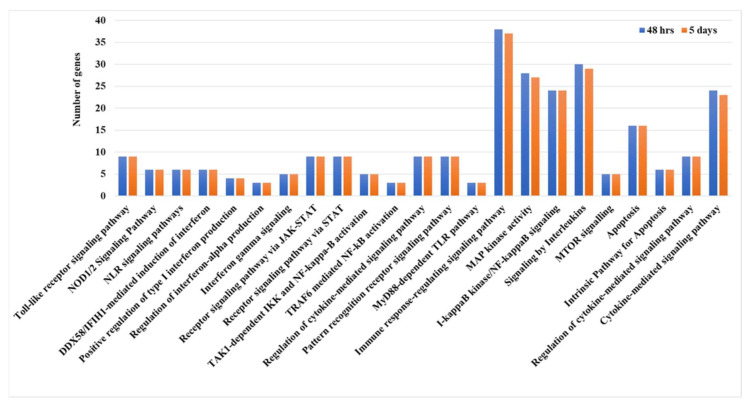
Pathway analysis of significantly enriched kinases whose substrates were differentially expressed phosphoproteins at 48 h and 5 days post-infection in duck lung tissues infected with the HPAI H5N1 virus.

High-resolution phosphoproteomic analysis uncovers important host kinases, their substrates, and signaling pathways that are modulated during avian influenza virus infection in ducks. In this study, phosphoproteomic profiling of duck lung tissue was performed at three different time points post-infection, and the top ten kinases enriched at each interval were identified. Kinases such as *CHAK1*, *CLK3*, *SRPK3*, *PKCD*, *HASPIN*, *SRPK2*, *PASK*, *PKCA*, *BMPR2*, *CHAK2*, and *SRPK1* showed enrichment at various stages after infection (Figure 4). These significantly enriched kinases were then used for pathway analysis, which revealed activation of several key signaling pathways, including toll-like receptor signaling, DDX58/IFIH1-mediated interferon-alpha/beta induction, NOD1/2 and NLR signaling, I-kappaB kinase/NF-kappaB signaling, JAK-STAT receptor signaling, regulation of interferon-alpha production, apoptosis, MyD88-dependent toll-like receptor signaling, cytokine-mediated signaling, interleukin signaling, TAK1-dependent IKK and NF-kappaB activation, TRAF6-mediated NF-kB activation, MAP kinase activity, and mTOR signaling in duck lung tissue during HPAIV infection (Table 1 and Figure 5). These pathways were also identified in previous integrated transcriptomic and proteomic analyses, confirming their activation in duck tissues during influenza infection. To pinpoint the main hub genes involved, an integrated protein–protein interaction network was constructed, highlighting *STAT1*, *DDX58 (RIG-I)*, *MYD88*, *NFKBIA*, *NFKB1*, *IRF7*, *SOCS3*, *ACTB*, *TLR4*, *TLR7*, *IL-6*, *CASP1*, and *CASP8* as central genes in disease pathogenesis and immune response in ducks (Table 2).

## 3. Discussion

### 3.1. Host Innate Immunity Against Influenza Infection in Ducks

Ducks rely on a highly developed innate immune system to defend against highly pathogenic avian influenza virus infection. Understanding these innate immune responses at a systems level is crucial for designing effective preventive and therapeutic strategies for avian influenza in birds. Advanced genomic approaches—including transcriptomics, proteomics, and phosphoproteomics—enable comprehensive analysis of global changes in the innate immune system and help clarify the molecular mechanisms underlying host defense and viral disease progression [8,9,10]. Our comprehensive network analysis integrating transcriptomic and proteomic data indicates a pronounced activation of multiple innate immune signaling pathways in the lungs of ducks infected with HPAIV. Specifically, the data highlight activation of RIG-I-like receptor, toll-like receptor, NOD-like receptor (NLR), NF-κB signaling, and JAK/STAT signaling pathways. Phosphoproteomic profiling independently validated the activation states of these pathways, reinforcing the findings derived from the integrated network clusters. Together, these analyses corroborate, at the computational level, the central role of the RIG-I-like receptor, toll-like receptor, NLR, NF-κB, and JAK/STAT signaling mechanisms in modulating the host response to HPAIV in duck lung tissue.

Host recognition of viral pathogen-associated molecular patterns (PAMPs) is primarily mediated by pattern recognition receptors (PRRs), including toll-like receptors (TLRs), retinoic acid-inducible gene I (RIG-I)-like receptors (RLRs), and nucleotide-binding oligomerization domain (NOD)-like receptor family pyrin domain-containing 3 (NLRP3). Engagement of these PRRs initiates signaling cascades that lead to the production of type I interferons (IFNs), pro-inflammatory cytokines, and chemokines, which orchestrate the antiviral immune response [21]. Toll-like receptors TLR3 and TLR7 act as intracellular sensors that recognize avian influenza viral RNA, initiating immune signaling through adaptor proteins such as TRIF and MYD88. This signaling cascade activates transcription factors, including interferon regulatory factor 7 (IRF7) and nuclear factor-κB (NF-κB), which stimulate the production of type I interferons and pro-inflammatory cytokines [22,23]. Specifically, in geese infected with H5N1 HPAIV, activation of the TLR7-MyD88-dependent pathway has been demonstrated to induce interferon-stimulated genes [22]. Furthermore, transcriptomic and proteomic analyses of duck lungs infected with H5N1 reveal pronounced activation of TLR7-MyD88 signaling, highlighting its key role in the early antiviral innate immune response [13,24,25].

Retinoic acid-inducible gene I (RIG-I) acts as a crucial pattern recognition receptor during influenza virus infection by detecting the viral 5′-triphosphate RNA. Upon recognition, RIG-I undergoes conformational changes that expose its caspase activation and recruitment domains (CARDs). These CARDs are subsequently ubiquitinated by the interferon-inducible E3 ubiquitin ligase tripartite motif-containing protein 25 (TRIM25) [26]. This modification facilitates RIG-I’s interaction with the mitochondrial antiviral signaling adaptor protein (MAVS), which triggers downstream phosphorylation and activation of transcription factors, including interferon regulatory factor 7 (IRF7) and nuclear factor kappa B (NF-κB). These factors induce the expression of type I interferons and pro-inflammatory cytokines and chemokines, orchestrating the antiviral immune response [11,27]. Notably, the presence of RIG-I in ducks contributes to their enhanced clearance of influenza viruses, whereas its absence in chickens is considered a factor for their increased susceptibility [28]. Further studies in other birds, such as domestic geese, Muscovy ducks and pigeons, confirm the conservation of this pathway, emphasizing its significance across anseriform and other birds in mediating disease outcomes [29,30,31,32].

NOD-like receptors (NLRs) play a pivotal role in the innate immune recognition of influenza virus in birds by assembling multiprotein inflammasome complexes, typically consisting of an NLRP protein, the adaptor ASC, and procaspase-1. Upon activation, caspase-1 mediates the cleavage of pro-IL-1β and pro-IL-18, facilitating the secretion of mature IL-1β and IL-18, which amplify the inflammatory response [10]. Transcriptomic studies show that the NLRC5 gene is upregulated in chicken lungs in response to highly pathogenic avian influenza virus (HPAIV) H5N1 infection, although the expression patterns may differ across species [15]. In ducks, NLRP3 expression is notably high in the pancreas, moderate in the trachea, and relatively low in the lung, but proteomic and phosphoproteomic data corroborate the activation of NLR pathways in duck lungs during HPAIV infection, underscoring their functional relevance in disease pathogenesis [24,25,33].

### 3.2. Type I Interferon Receptor Signaling Pathway and Expression of Interferon-Stimulated Genes

Activation of the aforementioned immunological pathways leads to the induction of interferons (IFNs), pro-inflammatory cytokines, and chemokines, which collectively initiate robust antiviral responses. Type I and II IFNs exert their effects by binding to their respective receptors—IFNAR1/IFNAR2 for type I and IFNGR1/IFNGR2 for type II—on the surface of the same or neighboring cells. The ligand–receptor interaction prompts dimerization of receptor subunits, consequently activating the Janus kinase–signal transducer and activator of transcription (JAK-STAT) signaling cascade. This activation culminates in the formation of the IFN-stimulated gene factor 3 (ISGF3) complex, a trimeric transcription factor consisting of STAT1, STAT2, and interferon regulatory factor 9 (IRF9), which drives the transcription of a broad array of interferon-stimulated genes (ISGs). Consistent with this established mechanism, integrated multi-omics analyses of HPAIV-infected duck lungs have confirmed the activation of interferon signaling, chemokine production, and JAK-STAT pathway activity. Activation of interferon signaling and JAK/STAT signaling pathways was reported in ducks infected with the HPAI H5N1 virus [17,24,34,35,36]. Expression of IFN-stimulated genes in response to HPAIV infection, including *MX1*, *IFITM5*, *OAS1*, *RSAD2*, *SOCS1*, *SOCS3*, *STAT1*, *STAT3*, *STAT5B*, *STAT6*, *IFIT5*, *IL10* and *PKR*, was observed in the lung tissues of ducks. Several of these genes have been implicated in influenza virus infection. The previous literature suggests that the expression of several of these genes is implicated in antiviral immunity to influenza virus infection [13,15,16,17,37,38,39,40].

### 3.3. Hub Genes Responsible for Disease Pathogenesis in Duck

Our investigation into the host response to avian influenza virus (AIV) infection supports the critical position of *STAT1*, *DDX58 (RIG-I)*, *MYD88*, *NFKBIA*, *NFKB1*, *IRF7*, *SOCS3*, *ACTB*, *TLR4*, *TLR7*, *IL-6*, *CASP1* and *CASP8* genes as a master regulatory hub in avian antiviral immunity.

STAT1 (Signal Transducer and Activator of Transcription 1) occupies a hub position in JAK/STAT-mediated signaling pathways; upon IFN binding, JAK kinases phosphorylate STAT1, leading to its dimerization and nuclear translocation to induce IFN-stimulated gene (ISG) expression, mediate interferon (IFN) signaling, apoptosis and antiviral defense mechanisms [41]. Ducks and wild waterfowl typically demonstrate a more modulated and localized STAT1 response compared to domestic chickens, balancing effective viral suppression with avoidance of excessive immunopathology [11,13,24,25]. Recent CRISPR and knockout studies in chickens further reveal that impairment of the STAT1 axis, such as with IFN receptor or STAT1 deletion, results in profound susceptibility—marked by higher viral burden and severe clinical outcomes [42]. Viral evasion mechanisms, such as influenza PB2 protein-mediated suppression of STAT1 activation, highlight the importance of maintaining robust STAT1 signaling for effective host defense [43]. Targeted nutritional or immunomodulatory approaches that boost STAT1 activity provide a practical path to enhancing antiviral defense and resilience in poultry exposed to avian influenza. Specifically, agents that promote STAT1 phosphorylation or counteract viral suppression of STAT1 are promising candidates for antiviral and immune modulation strategies [44].

DDX58 (RIG-I) is a key pattern recognition receptor that detects avian influenza virus (AIV) and initiates antiviral responses, particularly in respiratory and intestinal epithelial cells, which are primary sites of AIV replication [45,46,47]. RIG-I can act as an antiviral effector protein by directly binding to incoming IAV viral RNA [48]. Ducks express DDX58 and mount a robust but tempered antiviral response, while chickens lack DDX58 expression and rely on alternative pathways, making them more susceptible to severe pathology [11,12,24]. The NS1 protein of HPAI strains directly binds viral RNA to prevent DDX58 recognition and interacts with TRIM25 to disrupt the ubiquitination required for DDX58 activation, effectively suppressing the host antiviral response [49,50,51]. Synthetic RIG-I agonists have been developed to specifically activate RIG-I, leading to enhanced antiviral immunity and protection against viral infections, including influenza [52,53].

MYD88 serves as the adaptor for most TLRs, integrating signaling from TLR7, IL-1β, and IL-18 receptors and activating key downstream pathways (NF-κB, IRF1/5/7, MAPKs), vital for early host defense and inflammatory cytokine production [54]. Ducks show a more controlled MYD88 activation profile, with effective viral clearance and limited immunopathology. Ducks upregulate antiviral genes (e.g., IFN-stimulated genes) but limit pro-inflammatory cytokine expression, possibly due to evolutionary adaptations in MYD88 signaling kinetics and regulation [19,22,55,56]. MYD88 also mediates IL-1β and IL-18 receptor signaling, integrating TLR and inflammasome pathways in avian species [57]. This positions MYD88 as a central integrator of multiple danger signals during AIV infection, shaping the magnitude and quality of the immune response. MYD88 is essential for the immune-enhancing effects of several vaccine adjuvants, especially squalene emulsion-based adjuvants (e.g., MF59, AS03) used in influenza vaccines. These adjuvants depend on MYD88 signaling to boost antibody responses and dendritic cell maturation, though most mechanistic studies are in mammalian models [58]. In chickens and other birds, there are currently no studies directly using MYD88 as a therapeutic or vaccine agonist/antagonist in avian influenza infection.

The position of NFKBIA as a central regulatory hub that governs the balance between protective antiviral immunity and pathological inflammation in avian species [59,60]. The species-specific differences in NFKBIA regulation between chickens and ducks offer valuable insights for therapeutic development. In ducks and resistant chicken lines, association with robust NFKBIA resynthesis following initial degradation effectively terminates NF-κB signaling and prevents excessive inflammation. Conversely, in susceptible chickens infected with HPAI H5N1, this feedback mechanism appears compromised, with delayed NFKBIA reaccumulation contributing to the cytokine storm and immunopathology that define severe avian influenza [36,61,62,63]. Small molecule inhibitors of IKKβ, such as SAR113945, offer a more specific approach to maintain NFKBIA stability and suppress pathological NF-κB activation [64]. Controlled, transient NFKBIA degradation following vaccination could potentially amplify and shape the immune response without triggering pathological inflammation [65].

NFKB1 acts as a master regulator of the avian immune response to influenza infection, functioning both as a signaling component and an intrinsic inhibitor. In avian hosts, NFKB1 activation starts with the phosphorylation and partial proteasomal processing of p105 to produce mature p50 subunits, which then form transcriptionally active dimers with RelA (p65) [66]. Ducks show more efficient conversion of p105 to p50 during early infection, followed by rapid upregulation of p105 synthesis to terminate signaling—a pattern linked to their tolerance of AIV infection. Resistant chicken lines and ducks maintain higher basal levels of p105, which may help them modulate inflammatory responses more effectively than susceptible commercial poultry lines [36,59,67]. Additionally, several natural compounds have been reported to enhance p105 stability during HPAI infection, reducing pathological inflammation without fully suppressing antiviral responses [68]. Adjuvants such as AS03 have been shown to activate innate immune pathways, including NF-κB signaling, and enhance antibody responses to H5N1 vaccines, leading to improved immune activation and cross-protection against heterologous strains [69].

In avian hosts, IRF7 activation occurs via two main pathways: the endosomal TLR7/MyD88 pathway and the cytosolic RIG-I/MAVS pathway. Previous studies confirm that IRF7 acts as a central regulator of innate antiviral defense in chickens. IRF7 deficiency leads to reduced expression of antiviral interferons and interferon-stimulated genes (ISGs), resulting in enhanced viral replication in knockout cells, highlighting IRF7’s critical role in controlling virus spread [70,71]. Overexpression of IRF7 in chicken fibroblast cells promotes antiviral gene expression, but excessive activation may also increase cell death, potentially affecting disease severity [72]. Studies suggest that modulating IRF7 activity—either by agonists to boost immunity or antagonists to prevent excessive inflammation—could be a viable strategy for developing novel therapeutics or vaccines against avian influenza [70,72]. Research has shown that specific microRNAs (such as gga-miR-34c-3p) are differentially expressed during AIV infection and may modulate immune responses, including those involving IRF7, indicating their potential as targets for RNA-based therapeutics. Recent studies provide strong evidence for the identification of avian-specific microRNAs that regulate IRF7 expression and their potential as targets for RNA-based therapeutics to modulate the interferon response in avian influenza infection [73,74].

Suppressor of cytokine signaling 3 (SOCS3) is emerging as a critical negative regulator of cytokine signaling in avian species, playing a pivotal role in modulating inflammatory and antiviral pathways during avian influenza virus (AIV) infection. SOCS3 primarily functions by inhibiting JAK-STAT signaling cascades, thus regulating the intensity and duration of cytokine responses to prevent excessive inflammation and tissue damage [75]. Ducks tend to fine-tune SOCS3 induction to optimize antiviral immunity with minimal inflammatory injury, whereas susceptible birds often exhibit dysregulated SOCS3 expression, correlating with heightened cytokine storms and adverse outcomes [22,24,76]. Agents that increase SOCS3 expression (such as apocynin) have been shown to ameliorate immunopathology and improve survival in avian and mammalian models, suggesting SOCS3 agonists could be used to control excessive inflammation in avian influenza [77]. Modulating SOCS3 expression is being investigated as a strategy to balance immune responses—either by boosting antiviral immunity (via SOCS3 inhibition) or by preventing immunopathology (via SOCS3 induction)—in both vaccine and therapeutic contexts [78].

Upon AIV infection, robust upregulation of TLR4 expression is observed in avian respiratory and immune tissues, including lung, trachea, and spleen [79]. Functionally, TLR4 stimulation activates both MyD88-dependent and MyD88-independent (TRIF) pathways, inducing NF-κB activation, type I interferon production, and pro-inflammatory cytokines such as TNF-α, IL-1β, and IL-6 that restrict viral replication and recruit immune effectors, thus limiting virus spread and pathology [79,80]. Studies demonstrate that TLR4 agonists (such as LPS and novel ligands) can be used as adjuvants in poultry vaccines to enhance early antiviral responses [81]. TLR4 antagonists have been investigated to mitigate excessive inflammation and tissue damage in severe infections. For example, blocking TLR4 signaling can reduce cytokine storms and acute lung injury in influenza models, suggesting a therapeutic role in controlling hyperinflammation [82].

TLR7 resides in endosomal compartments of immune cells, such as plasmacytoid dendritic cells (pDCs) and macrophages, where it triggers MyD88-dependent signaling cascades, culminating in the production of type I interferons (IFN-α/β) and pro-inflammatory cytokines [22]. Resistant birds, such as ducks and certain chicken breeds, show robust early TLR7 induction, correlating with strong interferon and ISG responses, while susceptible lines exhibit blunted TLR7 signaling, higher viral loads, and increased mortality [22,83,84]. TLR7 ligands (including imiquimod and resiquimod) reduced AIV shedding and enhanced host immunity, with gene expression analysis confirming upregulation of IFN-α and other cytokines [74,83,85].

IL-6 plays a dual role, promoting both protective immunity and immunopathology depending on the magnitude and duration of its production. IL-6 promotes the acute phase response, enhances B-cell differentiation for antibody production, and facilitates the recruitment and activation of neutrophils and macrophages to infection sites, contributing to protective immunity [86,87]. Excessive IL-6 production in HPAI H5N1 infections is a key driver of the “cytokine storm,” leading to tissue damage and organ failure in fatal cases [88,89]. IL-6 levels are significantly higher in the lungs of chickens infected with HPAI H5N6 compared to ducks, highlighting the differential immune responses between species [90]. The strong link between IL-6 and disease severity has made it a focus for targeting IL-6 or its signaling pathways, underscoring its dual role as both a biomarker and a therapeutic target in influenza and other inflammatory diseases. Anti-inflammatory drugs such as monoclonal antibodies (e.g., tocilizumab) and JAK inhibitors (e.g., oclacitinib) have been studied for their ability to reduce harmful inflammation in viral infections, including influenza [91,92,93]. However, further clinical trials are needed to determine the optimal timing, dosing, and patient selection for IL-6-targeted therapies in managing influenza infections [87].

ACTB, which encodes β-actin, is a fundamental cytoskeletal protein involved in maintaining cell structure, intracellular trafficking, and facilitating immune cell functions. During avian influenza virus (AIV) infection, emerging evidence demonstrates that ACTB and associated cytoskeletal elements play crucial roles in viral entry, replication, assembly, and host immune responses in birds [94,95]. Moreover, influenza viral components, such as the PA subunit of the RNA-dependent RNA polymerase (RdRp), interact directly with β-actin, suggesting the viral hijacking of cytoskeleton proteins to optimize infection efficiency [96]. Alterations in actin stability and phosphorylation status during infection have also been linked to increased viral replication and spread [97].

Caspases, particularly CASP1 and CASP8, play pivotal and interconnected roles in determining the outcome of avian influenza virus (AIV) infection in birds by regulating cell death and inflammation. NLRP3 inflammasome activation leads to CASP1 cleavage and the maturation of IL-1β and IL-18, which are critical for antiviral immunity and inflammation [98]. CASP8 activation during AIV infection enhances antiviral responses by cleaving CYLD and blocking TAK1 and RIG-I while also preventing necroptosis by cleaving RIPK1 and RIPK3 [92]. Studies show that AIV infection can activate both apoptosis and necroptosis pathways, with CASP8 playing a central role in regulating the balance between these processes [99]. VX-765 (a selective caspase-1 inhibitor) treatment in murine models of severe influenza significantly reduced pro-inflammatory cytokine levels, immune activation, and mortality, highlighting its potential as a therapeutic agent for severe viral infections [100]. In summary, previous research strongly supports that most of the hub genes identified in our network analysis play crucial roles in the innate immune response against influenza infection, and some of these genes may serve as potential therapeutic or vaccine agonist/antagonist targets in avian influenza infection in birds. However, further biological validation through in vitro or in vivo experiments is needed to clarify the critical functional roles of these genes in the innate immune mechanism of ducks.

## 4. Materials and Methods

### 4.1. Experimental Infection of Ducks

Six-week-old healthy domestic ducks, confirmed to be seronegative for avian influenza virus (AIV), were used in this study. The animal experiments were approved by the Institutional Animal Ethics Committee of ICAR-NIHSAD (approval no. 68/IAEC/HSADL/12, dated 11 May 2012), and all procedures were carried out in a biosafety level 3 containment facility at the ICAR National Institute of High-Security Animal Diseases, Bhopal, India. The ducks were divided into four groups (n = 5 per group). Three groups were inoculated intranasally with 10^6^ EID_50_ of H5N1 virus (A/duck/India/02CA10/2011/Agartala), while one group served as the control and received PBS. The birds were monitored daily for clinical signs. Lung tissues were collected from five birds in each infected group at 12 h, 48 h, and 5 days post-infection and from the control group at 12 h post-inoculation. The tissues were snap-frozen in liquid nitrogen and stored at −80 °C until RNA and protein extraction. AIV infection in lung tissues was confirmed by virus isolation in embryonated chicken eggs and RT-PCR.

### 4.2. RNA Sequencing

The stored lung samples were used for RNA extraction using the RNeasy kit (QIAGEN, Hilden, Germany). RNA quality and quantity were checked using NanoDrop (Thermofisher, Wilmington, DE, USA) and Bioanalyser (Agilent Technologies, Santa Clara, CA, USA). From each post-inoculation condition, the three highest-quality samples out of five were selected for sequencing. For paired-end sequencing, mRNA libraries were prepared and sequenced on an Illumina Hiseq2500 platform (Illumina, San Diego, CA, USA). RNA sequence genome mapping and read count procedures were performed using the GALAXY public server [101] (https://usegalaxy.org/). Quality control of the raw sequencing reads was carried out using the FastQC v0.11.9 program. Trimmomatic v0.39 software was used for quality filtering of the short reads [102]. The genome mapping of both control and infected RNA-seq sample datasets against the duck genome (NCBI Genome assembly: GCF_047663525.1) was conducted using hierarchical indexing for spliced alignment of transcripts 2 (HISAT2) spliced aligner [103]. The SAM/BAM file generated from genome mapping and the GTF file downloaded from the NCBI database (https://ftp.ncbi.nlm.nih.gov/genomes/all/GCF/047/663/525/GCF_047663525.1_IASCAAS_PekinDuck_T2T/, accessed on 5 July 2025) were used as inputs to the htseq-count tool [104]. Raw read counts were normalized using the Trimmed Mean of M-values (TMM) method, and differential expression analysis was conducted in edgeR using a generalized linear model (GLM). Genes showing significant differential expression between conditions were identified based on a false discovery rate (FDR) < 0.05 and an absolute log fold change (logFC) > 1.5 [105,106].

### 4.3. Protein Sample Preparation, LC-MS and Bioinformatics Analysis

The stored lung tissue was cut into small pieces, and 650 μL of SDS protein extraction lysis buffer [0.1% SDS (Invitrogen, Waltham, MA, USA); 50 mM NH_4_HCO_3_ (Sigma, Saint Louis, MO, USA); 1x Complete™ Protease Inhibitor Cocktail (Roche Diagnostics GmbH, Mannheim, Germany)] was added. The tissue lysate was incubated on ice for 90 min to ensure complete protein lysis. The lysate was then centrifuged at 20,000× *g* for 60 min at 4 °C, and the supernatant was collected. The supernatants were immediately snap-heat-treated at 56 °C for 30 min in a dry bath to inactivate HPAIV H5N1 in the protein extracts. All heat-treated samples were stored at −80 °C for mass spectrometry analysis.

The samples were subjected to trypsin digestion (enzyme:substrate 1:20) at 37 °C overnight. After trypsin digestion, the samples were labeled with Tandem Mass Tag™ (TMT) (Thermo Scientific™, Waltham, MA, USA) and then used for LC-MS/MS analysis in an Orbitrap Fusion Tribrid mass spectrometer (Thermo Scientific™) to determine the expression of the total quantitative proteome and phosphoproteome (phosphopeptide enrichment using TiO_2_ beads). The data from both the total proteome and phosphoproteome datasets were analyzed using the Proteome Discoverer 2.1 software suite (Thermo Scientific™). The data were searched using the SequestHT algorithm. The reference duck proteome dataset was downloaded from the NCBI database.

### 4.4. Integration of Multi-Omics Data into Protein–Protein Interaction Networks

The integration of multi-omics data into protein–protein interaction (PPI) networks was carried out using Cytoscape (version 3.9) with the following installed applications: STRING App for PPI network retrieval and analysis [107], MCODE for cluster detection [108], and ClueGO for pathway enrichment analysis [109]. The PPI network was built using the STRING database, incorporating differentially expressed genes (DEGs) and proteins (DEPs) with a disease-specific query and a minimum confidence score threshold of 0.7. Transcriptomic and proteomic datasets were systematically integrated into the PPI network. mRNA expression data were imported by matching gene identifiers in the input file to corresponding nodes in the network, with log2 fold change values and associated *p*-values mapped as node attributes. Similarly, protein abundance data were incorporated using the same identifier matching approach. Multi-omics visualization was achieved through strategic node and edge styling within Cytoscape’s Style panel. Node fill color was mapped to mRNA expression levels using a continuous red–blue gradient, where red indicated upregulation and blue represented downregulation. Node size was proportional to protein abundance changes, scaled between 10 and 80 pixels based on log2 fold change values (Figure 1).

Key functional modules were identified using MCODE v1.0, with parameters set to a degree cutoff of 5 and node scoring based on mRNA expression changes (Figure 2). The resulting clusters were subjected to pathway enrichment analysis through ClueGO, where selected nodes from significant clusters were analyzed against KEGG and Reactome databases with a *p*-value cutoff of 0.05. This integrated approach enabled the identification of biologically relevant pathways and signaling cascades underlying the multi-omics patterns observed in the network (Figure 3).

### 4.5. Phosphoproteome-Driven Kinase Activity Inference

Kinase Enrichment Analysis 3 (KEA3) (https://maayanlab.cloud/kea3/, accessed on 5 December 2025) identifies upstream kinases whose putative substrates are overrepresented in a list of differentially phosphorylated proteins [110]. The KEA3 algorithm uses a rank-based enrichment approach, testing whether known substrates of each kinase are significantly enriched at the extremes of the ranked list, employing statistical methods similar to Gene Set Enrichment Analysis. Kinases with significant enrichment scores (*p*-value < 0.05) are considered for further analysis (Figure 4). To provide biological context and validate results, significantly enriched kinases are mapped to known signaling pathways by integrating with the KEGG and Reactome databases using ClueGO, enabling the identification of activated or inhibited signaling cascades and facilitating biological interpretation of the observed phosphoproteomic changes in the experimental system (Figure 5).

## 5. Conclusions

The integration of transcriptomic and proteomic analyses reveals strong activation of several innate immune signaling pathways in the lungs of ducks infected with HPAIV. Notably, the RIG-I-like receptor, toll-like receptor, NOD-like receptor, NF-κB, and JAK/STAT pathways are prominently engaged, leading to the upregulation of interferon-stimulated genes and the establishment of a robust antiviral response in HPAI H5N1-infected lung tissue. Phosphoproteomic data independently confirm the activation of these pathways, supporting the network-based findings. Key regulatory proteins—including STAT1, NFKB1, and others—emerge as central hubs in the duck’s antiviral defense and may represent promising targets for future therapeutics or vaccine development. These insights lay the groundwork for innovative strategies to enhance animal health, safeguard food security, and reduce the risk of global pandemics by identifying novel intervention points for avian influenza control.

## Figures and Tables

**Table 1:** Pathway analysis of significantly enriched kinases whose substrates were differentially expressed phosphoproteins at 12 h post-infection in duck lung tissues infected with the HPAI H5N1 virus.

Sl. No.	Pathways	Number of Genes	*p* Value
1	Regulation of cytokine-mediated signaling pathway	9	0.00086
2	Pattern recognition receptor signaling pathway	9	0.0034
3	Toll-like receptor signaling pathway	9	0.00018
4	MyD88-dependent toll-like receptor signaling pathway	3	0.00598
5	Immune response-regulating signaling pathway	35	1.2 × 10^−12^
6	MAP kinase activity	27	1 × 10^−16^
7	I-kappaB kinase/NF-kappaB signaling	24	3.9 × 10^−11^
8	Receptor signaling pathway via JAK-STAT	9	0.00221
9	Regulation of interferon-alpha production	3	0.01338
10	Regulation of interleukin-2 production	4	0.02069
11	Positive regulation of interferon-alpha production	3	0.00672
12	Negative regulation of viral genome replication	3	0.06207
13	Interleukin-6-mediated signaling pathway	3	0.00258
14	Interleukin-1-mediated signaling pathway	8	7.6 × 10^−9^
15	Receptor signaling pathway via STAT	9	0.00285
16	Apoptosis	16	2.4 × 10^−8^
17	Intrinsic pathway for apoptosis	6	0.00021
18	MTOR signaling	5	0.00042
19	NOD1/2 signaling pathway	6	2.1 × 10^−5^
20	NLR signaling pathways	6	0.00026
21	DDX58/IFIH1-mediated induction of interferon-alpha/beta	6	0.00178
22	TAK1-dependent IKK and NF-kappa-B activation	5	0.00015
23	Interleukin-17 signaling	20	5.7 × 10^−20^
24	Signaling by interleukins	29	2.1 × 10^−10^
25	TRAF6 mediated NF-kB activation	3	0.00672

**Table 2:** Hub genes identified in duck PPI networks based on degree centrality measure.

Rank	12 h	48 h	5 Days
1	*STAT1*	*STAT1*	*STAT1*
2	*NFKB1*	*IFNG*	*MYD88*
3	*MYD88*	*TLR7*	*TLR4*
4	*IL6*	*TLR4*	*CASP1*
5	*TLR4*	*CXCL8*	*TLR7*
6	*IFNG*	*DDX58*	*NFKB1*
7	*DDX58*	*CCL5*	*ACTB*
8	*CASP8*	*SOCS3*	*DDX58*
9	*TLR3*	*ACTB*	*NFKBIA*
10	*ACTB*	*CASP1*	*IRF7*

## Data Availability

The duck RNA-seq data generated in this study have been publicly deposited in the National Center for Biotechnology Information (NCBI) Sequence Read Archive (SRA) under the accession number PRJEB41063. However, the raw proteomics and phosphoproteomics datasets supporting the conclusions of this article will be made available by the authors on request.
